# FluReF, an automated flu virus reassortment finder based on phylogenetic trees

**DOI:** 10.1186/1471-2164-12-S2-S3

**Published:** 2011-07-27

**Authors:** Alisa Yurovsky, Bernard M E Moret

**Affiliations:** 1 Department of Computer Science, EPFL (Swiss Federal Institute of Technology), Lausanne, CH-1015, Switzerland

## Abstract

**Background:**

Reassortments are events in the evolution of the genome of influenza (flu), whereby segments of the genome are exchanged between different strains. As reassortments have been implicated in major human pandemics of the last century, their identification has become a health priority. While such identification can be done “by hand” on a small dataset, researchers and health authorities are building up enormous databases of genomic sequences for every flu strain, so that it is imperative to develop automated identification methods. However, current methods are limited to pairwise segment comparisons.

**Results:**

We present FluReF, a fully automated flu virus reassortment finder. FluReF is inspired by the visual approach to reassortment identification and uses the reconstructed phylogenetic trees of the individual segments and of the full genome. We also present a simple flu evolution simulator, based on the current, source-sink, hypothesis for flu cycles. On synthetic datasets produced by our simulator, FluReF, tuned for a 0% false positive rate, yielded false negative rates of less than 10%. FluReF corroborated two new reassortments identified by visual analysis of 75 Human H3N2 New York flu strains from 2005–2008 and gave partial verification of reassortments found using another bioinformatics method.

**Methods:**

FluReF finds reassortments by a bottom-up search of the full-genome and segment-based phylogenetic trees for candidate clades—groups of one or more sampled viruses that are separated from the other variants from the same season. Candidate clades in each tree are tested to guarantee confidence values, using the lengths of key edges as well as other tree parameters; clades with reassortments must have validated incongruencies among segment trees.

**Conclusions:**

FluReF demonstrates robustness of prediction for geographically and temporally expanded datasets, and is not limited to finding reassortments with previously collected sequences. The complete source code is available from http://lcbb.epfl.ch/software.html.

## Introduction

Influenza (the “flu”) is an RNA virus with an extremely high mutation rate [1-3] that causes fever and respiratory problems in humans and other animals. It is responsible for half a million human deaths every year [4]. Flu populations typically experience a seasonal bottleneck event, as host-to-host transmission in the temperate regions drops to very low levels during the warm season. According to the source-sink hypothesis [2], new strains of viruses are seeded from a flu reservoir in the tropics, called the source, and spread seasonally to the temperate zones, called sinks [5-7], thus creating multiple coexisting generations of flu strains in the temperate regions [8].

The genome of the flu virus is composed of eight *segments.* Reassortment of segments among flu virus strains, i.e., mixing of segments from one or more strains to produce new strains, is a frequent event [9-11]. Strains resulting from such reassortments have been responsible for two of the three great pandemics of the 20th century [12].

A large number of fully sequenced flu genomes recently became publicly available [13], but large amounts of data cannot be processed by the most widely used reassortment-finding techniques, as these involve human scrutiny of phylogenetic trees. In these methods, one constructs a phylogenetic tree based on the full genomes, as well as a tree based on each of the eight segments, for a total of nine trees; one then examines these trees, looking for strains that have segments on different branches of their respective trees [12,14]. While the visual inspection method is intuitive and logical, it is prohibitively time-consuming, and outright inapplicable when thousands of samples are to be examined.

A few methods have been proposed to process the flu data automatically. Rabadan *et al.*[11] postulated that, for any two strains, the Hamming distance between their respective first segments and the Hamming distance between their respective second segments should be equal (after normalization) in the absence of reassortment, while different distances should point to a reassortment. Niranjan *et al.*[10] used phylogenies, but considered distributions of phylogenetic trees for each segment, instead of the consensus tree. Their method enumerates the maximal bicliques on a bipartite graph of tree edges for the distributions of the two segments; these bicliques represent sets of mutually incompatible choices, indicating that the two segments may have had different evolutionary histories. Both methods are limited to detecting reassortments between collected and sequenced strains. While these approaches were used to detect meaningful reassortment events [11,15], they are not scalable to large datasets when all reassorted segments need to be identified, because they use pairwise comparisons which must then be manually aggregated.

### Our contribution

Our new, fully automated, flu reassortment finding algorithm, FluReF, embodies and parameterizes the structural observations used in visual reassortment finding. We describe an algorithm that examines the reconstructed phylogenetic trees of individual segments and of the full genome, selects candidate reassortment groups through a bottom-up search of the full phylogenetic tree, and confirms candidates that meet preset thresholds and cause demonstrated incongruencies among segment trees.

FluReF is designed to find all segments involved in all reassortment events in a dataset. The method is scalable, running in time quadratic in the number of full genome sequences. Furthermore, FluReF is not limited to finding reassortments among sequenced strains, as it searches for reassortments with ancestral source strains.

We also present a simple simulator for the evolution of flu genomes, in terms of point mutations and reassortment, and incorporating both strain isolation and bottleneck events. On data produced by this simulator, FluReF tuned for a 0% false positive rate has consistently demonstrated a 10% false negative rate. On sequence data from influenza databases, FluReF corroborated two new reassortments identified during our visual analysis of the 75 Human H3N2 New York flu strains from 2005–2008. FluReF demonstrated robustness of prediction with temporally and geographically expanded datasets. We obtained partial verification of reassortments found by Holmes *et al.*, who performed a phylogenetic analysis of 156 Human H3N2 New York flu strains from 1999–2004 [8].

## Results

We first describe the principles behind our method. Next, we present the FluReF algorithm, including the description of tunable parameters. We then present the results on various flu datasets from the state of New York. We conducted a visual analysis of a collection of 2005–2008 New York flu genomes, identifying two reassortments, and ran FluReF on this dataset. We then expanded the temporal and geographic scope of the data to test the robustness of FluReF by augmenting our dataset with (i) a large number of sequences from the same area (New York) from a prior year (2004) and (ii) sequence data from all over the United States. We then ran FluReF on another, unrelated flu dataset from Holmes *et al.*[8]. Finally, we experimented with a larger set of simulated sequences.

### FluReF: principles

FluReF exploits certain characteristics of phylogenetic trees of the flu genome. The trees produced from samples taken over a number of years in the same geographical location follow a well established pattern—sequences from the same year tend to cluster together, sometimes forming a clade with sequences from the year before or the year after [3]. Another common feature of localized phylogenetic trees is that sequences collected in earlier years tend to be closer to the root than those collected in later years, as they had less time to evolve away from the common ancestor at the root. In visual inspection methods [12,14], the exploration starts by examining the full genome tree, looking for individual sequences, or small groups of sequences, that do not fit these characteristics.

**Figure 1:**
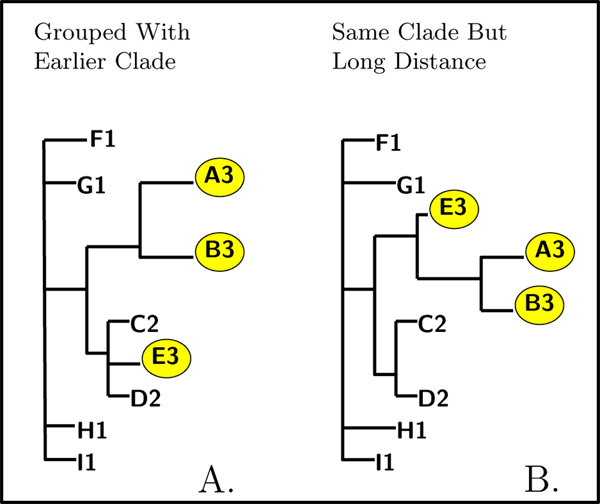
Separation from seasonal group

For example, we may find some sequences that are not grouped with the others from the same year, but with sequences sampled in an earlier year: Figure 1 (A) shows a toy example where clade E from year 3 is grouped together with the sample from year 2. Similarly, we may find some sequences that, while grouped together with the rest of the season, are separated from them by a significantly large distance: Figure 1 (B) shows clade E correctly grouped with the other samples from year 3, but at a significant evolutionary distance from them. In either case, sequences phylogenetically separated from their seasonal grouping are candidates for reassortment. We postulate that this genetic disparity is possible if a strain from the sampling year, the survivor of the previous bottleneck event, has reassorted some of its segments with a strain that re-emerged from the source population. We assume that the lower selective pressure in the source population results in slower evolutionary change, so that a re-emerging sample from the source population would be more genetically similar to the sink population from prior sampling years.

**Figure 2:**
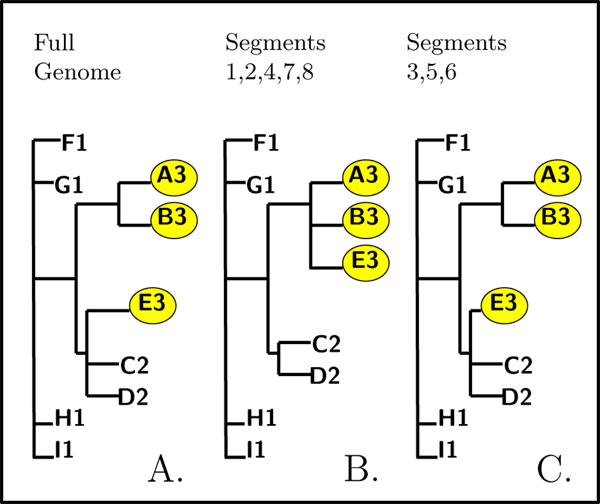
Reassortment search

To test for a reassortment, we examine the eight segment trees, searching for an isolated candidate clade. If the candidate clade remains isolated in all individual segment trees, the reason is unrelated to reassortment. One of the possible explanations is that such candidate strains infected the human host in a geographic area far away from the sampling area and thus have a somewhat different evolutionary history. If, however, the candidate clade is grouped together with the other samples from its season in some of the segments, but is isolated in others, we have identified a probable reassortment. Figure 2 shows a toy example with three sampled years. Segments in the isolated candidate clade E3 (3, 5, and 6) have come from the seasonal migration of the source strain, while the rest of the segments for E3 (1, 2, 4, 7, and 8) came from the local seasonal population.

### FluReF: algorithm

FluReF carries out an exhaustive bottom-up search of the phylogenetic tree reconstructed from the full genome sequences. As the search proceeds, various measures are checked to ensure that candidate reassortments satisfy parameter thresholds motivated by the visual inspection.

In the main loop of the algorithm, each leaf node (a single sequence) is considered if it was not already identified as part of a candidate reassortment. A candidate group is grown upward from the leaf, expansion terminating upon reaching the noise threshold—exceeding a tuneable parameter which dictates when the candidate group would encompass an unacceptably heterogeneous sample from different years.

**Figure 3:**
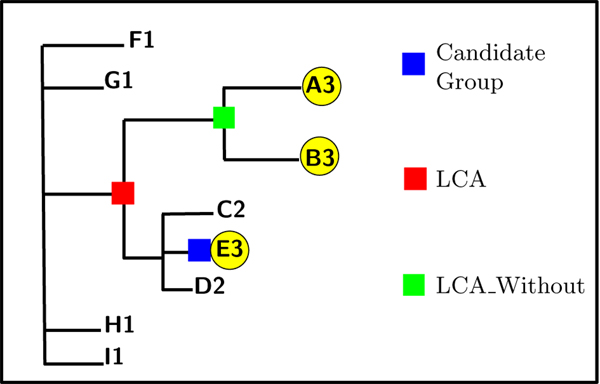
Candidate group (Blue/Black), LCA (Red/Dark Gray), LCA Without (Green/Light Gray)

Once a candidate group is identified, the Least Common Ancestor (LCA) is found for all leaves sampled in the year that contains the majority of sequences in this candidate group, as shown in Figure 3.

Next, the Least Common Ancestor excluding the candidate group (LCA_Without) is found. Various metrics for the path from the candidate group to the LCA_Without, via the LCA, are checked to ensure that the separation distance is nontrivial and that the three path has strong support.

In the visual reassortment search method, the path is examined to ensure that it contains several edges with very high confidence values as provided by the phylogenetic reconstruction software. In general, it is desirable to have a majority of edges on the path with reasonably high confidence values, generating trust in the existence of the candidate group separation. FluReF translates this intuition into several tuneable parameters which minimize the rate of false positives by ensuring that only paths with high confidence values from the phylogenetic reconstruction are considered in a reassortment search. During the visual reassortment search, the candidate group is assessed for its distance away from the rest of the season, compared to the rest of the tree. FluReF encompasses this observation with a couple separation parameters, tuned to ignore candidate groups with a trivial genetic separation from the rest of the season. For each candidate group which satisfies all parameters, the algorithm then attempts to find the analog of this candidate group in each of the individual segment trees. If a group is found in a segment tree, it is again checked against various parameter thresholds—typically lower than those used with the tree based on the full genome sequences, because the confidence values from the phylogenetic reconstruction software tend to be lower for individual segment trees. The candidate group is output as a reassortment if it is found to be isolated from the rest of the year sample in some segment trees, but is grouped with the rest of the year sample in other segment trees, pointing to different evolutionary histories. (Preference may be given to certain segments, as there is evidence that some segments are more commonly involved in reassortments than others [11].)

FluReF runs in at most quadratic time. The main loop traverses a tree, taking time proportional to the size of the tree, i.e., proportional to *n*, the number of leaves; if each leaf (strain) is considered as a separate candidate group, the main loop will iterate *n* times.

### Experiment 1: confirming visual inspection

**Figure 4:**
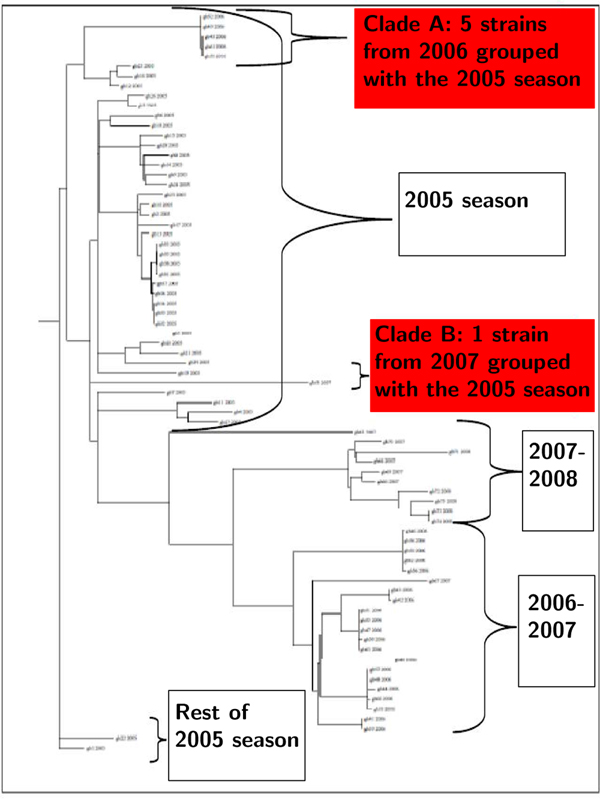
Full-genome phylogenetic tree for 75 Human H3N2 strains from New York, 2005–2008

We examined a dataset of 75 Human H3N2 strains collected between 2005 and 2008 in New York. The visual inspection of full-sequence and individual segment phylogenetic trees revealed two reassortments. Clade A from 2006, shown in Figure 4, was grouped separately from the rest of its season in the full genome tree, as well as in individual trees for segments 1, 2, 3, 5, and 6. Clade B from 2007, also shown in Figure 4, was grouped separately from the rest of its season in the full genome tree, and in individual trees for segments 3 and 4. We applied FluReF to this data set; it produced no false positives and output both Clades A and B as reassortment groups, with the same segments identified as in the visual analysis. This result confirms that FluReF properly applies the principles of the visual analysis of phylogenetic trees.

### Experiment 2: increasing the temporal scope

To test the robustness of FluReF, we augmented the dataset from Experiment 1 with human H3N2 strains sampled in 2004 from New York. The new data set thus contains 118 sequences—at the limit of what visual inspection can handle. FluReF run on this dataset returned the same output as on the unaugmented dataset used in Experiment 1, once again matching visual inspection results.

### Experiment 3: increasing the geographic scope

The inclusion of geographically separated strains can lead to the isolation of subgroups from their seasonal cohort and thus potentially cause false positive identifications. We augmented the dataset from Experiment 2 with the rest of the 2005–2008 human H3N2 strain sequences collected all over the United States. The resulting data set contains 180 sequences, beyond our ability to inspect visually. FluReF once again returned the same output as on the unaugmented dataset from Experiment 1, a reassuring result in that it was not misled by geographically isolated strains.

### Experiment 4: validating prior work

In 2005, Holmes *et al.* performed a phylogenetic analysis of 156 complete genomes of human H3N2 influenza A viruses collected between 1999 and 2004 from New York State and found several reassortment events between the various clades [8]. Aside from between-clade reassortments, which are currently not targeted by FluReF, Holmes *et al.* identified three reassortment groups. Run on the same data, FluReF confirmed one of these candidate reassortment groups: a small clade containing two strains from 1999: [GenBank:CY001120-27, GenBank:CY000989-96]; another candidate group was considered by the algorithm, but rejected due to low confidence scores. We have tuned the parameters of FluReF to be very conservative, so the absence of false positives and the occurrence of some false negatives are to be expected; a more sensitive tuning is possible, especially one that favors certain segments over others, a bias adopted by Holmes *et al.* in their analysis.

### Experiment 5: scaling

While the quadratic limit makes FluReF scalable in terms of runtime, care must be take to ensure that the accuracy of the algorithm does not suffer as the datasets increase. We performed a first scaling experiment, with a set of 420 simulated sequences containing a single reassortment event. FluReF found this reassortment, and reported no false positives.

## Methods

### External software and materials

All influenza A sequences were downloaded from the NCBI Influenza Virus Sequence database [13]. GenBank sample identifier strings were modified to include the year of sampling and a short unique identifier, to aid in the visual inspection of the phylogenetic trees. MAFFT, a multiple alignment program based on Fast Fourier transforms [16], was used to align the sequences for all experiments using real data. The RAxML web server [17] was used to reconstruct the phylogenetic trees for all real and synthetic datasets using the Maximum Likelihood approach.

### Tuning FluReF

Parameter thresholds were tuned on simulated data generated by our simulator (described farther down) to keep the number of false positives down to zero, while minimizing the number of false negatives. Tuning was done using two dozen small datasets of 40 strains each. With tuned parameters, FluReF, when run on these simulated datasets, finds no false positives (nonexistent reassortment) and fails to find 10% of the existing reassortments, for a 0% false positive rate and a 10% false negative rate.

### FluReF usage

FluReF input consists of nine files with phylogenetic trees reconstructed by RAxML from aligned sequences of viruses; these sequences should come from sampling, at regular intervals, of the flu genome within a well defined geographic area (or, of course, from simulations). The first eight files contain the trees for their respective segments, while the ninth file contains a tree reconstructed from full genome sequences for each virus strain. The output provides the identifier(s) of all viruses in groups that have undergone a reassortment. For each group, the names of the segments that participated in the reassortment are also output.

### Viral evolution simulator

In the absence of sufficient verified reassortment data, we needed a viral evolution simulator to produce synthetic data sets with known reassortments, so as to be able to test and tune our algorithm. We developed a very simple simulator, which incorporates some more recent theories on the flu evoltion, and used it to produce synthetic datasets that resulted in realistic phylogenies.

We begin the simulation by initializing the start source population. The start source population consists of any number of real virus sequences, downloaded from NCBI, preferably collected from the same geographic and temporal location. The input is separated into eight files, each containing aligned sequences of all source population viruses for their respective segments. The output consists of nine files with aligned sequences sampled at regular intervals from the sink population. The eight files each contain virus sequences for their respective segments, and the ninth file contains a full genome sequence for each virus strain, all in the format described for input to FluReF.

We model the viral evolution by maintaining two groups of viruses. The first group is kept at a stable size, to mimic the viral source in the tropics. The second group models a local virus sink; it expands every sampling interval and then contracts in a bottleneck event, to mimic the seasonal flu cycles. Instead of maintaining individual viral strains, we maintain populations of relatively small size. A population consists of up to one hundred viruses with an identical sequence, a simple way of modelling closely related strains or the viral quasispecies population.

We model point mutations using the Kimura two-parameter substitution model [18]. We introduce an operation we call “global parallel mutate,” which makes identical mutations in any number of populations. We apply this operations to all viral populations in source and sink to mimic conditions that make certain mutations more advantageous during a particular season, as well as to mimic the super-viral strains that rapidly spread through the world during a particular season. We also use a regular, “divergent” mutate operation, that makes unique mutations in each viral population and is responsible for individual variations between the populations.

We perform a reassortment between one population in the source and one population in the sink once per sampling period. The genetic transfer is unidirectional, as the genetic flow is thought to be unidirectional from the source to the sink regions. At the end of each sampling period, after the bottleneck event, we output a small, randomly selected sample of survivor sequences.

## Discussion

FluReF builds upon the visual inspection of reconstructed phylogenetic trees, which is the most commonly used and best accepted method for finding reassortments. However, whereas visual inspection is limited to datasets of a hundred samples, FluReF is designed to be scalable for very large data sets. Its running time is at most quadratic in the number of samples, so that analyses of datasets with tens or hundreds of thousands of samples can be carried out rapidly—the computational bottleneck is the reconstruction of the nine phylogenetic trees, not the search for reassortments.

The FluReF model does not tie us to searching for reassortments between pairs (or triplets) of sampled and sequenced strains in the same data set. This is very important, as it is it entirely possible that only one of the strains involved in a reassortment has been sampled.

While our results are promising, much work remains to be done. FluReF will benefit from extensive parameter tuning on real datasets with proven reassortments. Focusing the search on segments more likely to be involved in reassortments will both speed up the search and increase its sensitivity—our current implementation is quite conservative and favors specificity over sensitivity. Finally, reassortments between clades could also be sought by a similar approach, with its own set of parameters.

## Conclusions

The recent swine flu epidemic, along with discoveries of correlations between different circulating strains of flu, underscore the importance of genomic analyses for future influenza surveillance. Even reassortments in the same lineage can cause a severe outbreak and failure of vaccine coverage. As our databases of flu genomes increase very rapidly, computational approaches must be deployed to analyze the large volume of data and help identify candidate events, such as reassortments, that may pose new health threats. We developed FluReF, an automatic reassortment finder algorithm inspired by the visual identification approach. FluReF parameters were tuned on the synthetic datasets produced by our simple virus evolution simulator to yield no false positives; even at this very conservative setting, FluReF had very high sensitivity, with false negative rates consistently below 10%. With these parameter values, FluReF corroborated the two reassortments we found during a visual analysis of the 75 Human H3N2 New York strains from 2005–2008; it also demonstrated robustness of prediction with temporally and geographically expanded datasets, and verified some of the reassortments found using another bioinformatics method. FluReF is only as good as the data for which its parameters have been tuned; while flu databases accumulate ever more sequences, quality annotation of reassortments has been very limited to date. Any approach to reassortment finding based on statistics or machine learning will benefit from additional reference data.

## Competing interests

The authors declare that they have no competing interests.

## Authors contributions

AY wrote the algorithms and carried out the experiments. BM provided guidance and direction. Both authors worked on the manuscript.
